# Clinical and Molecular Characteristics and Antibacterial Strategies of Klebsiella pneumoniae in Pyogenic Infection

**DOI:** 10.1128/spectrum.00640-23

**Published:** 2023-06-21

**Authors:** Zhiyu Yang, Runmei Zhou, Yusheng Chen, Xiaotuan Zhang, Luogen Liu, Min Luo, Jinlin Chen, Kuilin Chen, Tong Zeng, Bin Liu, Yuan Wu, Jielite Huang, Zhuoran Liu, Jinglin Ouyang

**Affiliations:** a Department of Clinical Laboratory, Second Affiliated Hospital, Hengyang Medical School, University of South China, Hengyang, China; b Department of Pharmacy, Second Affiliated Hospital, Hengyang Medical School, University of South China, Hengyang, China; c Department of Pharmacy, Ningyuan County Hospital of Traditional Chinese Medicine, Ningyuan, Hunan, China; d Clinical Research Center, Second Affiliated Hospital, University of South China, Hengyang, China; e Key Laboratory of Medical Imaging and Artificial Intelligence of Hunan Province, Xiangnan University, Chenzhou, China; f Clinical Laboratory, Affiliated Nanhua Hospital, Hengyang Medical School, University of South China, Hengyang, China; g Department of Ultrasound Medicine, Second Affiliated Hospital, Hengyang Medical School, University of South China, Hengyang, China; Institut Pasteur

**Keywords:** Hypervirulent *Klebsiella pneumoniae*, classical *Klebsiella pneumoniae*, pyogenic infection, clinical and molecular characteristics, antibacterial strategies

## Abstract

Treatment of Klebsiella pneumoniae causing pyogenic infections is challenging. The clinical and molecular characteristics of *Klebsiella pneumoniae* causing pyogenic infections are poorly understood, and antibacterial treatment strategies are limited. We analyzed the clinical and molecular characteristics of K. pneumoniae from patients with pyogenic infections and used time-kill assays to reveal the bactericidal kinetics of antimicrobial agents against hypervirulent K. pneumoniae (hvKp). A total of 54 K. pneumoniae isolates were included, comprising 33 hvKp and 21 classic K. pneumoniae (cKp) isolates, and the hvKp and cKp isolates were identified using five genes (*iroB*, *iucA*, *rmpA*, *rmpA2*, and *peg-344*) that have been applied as hvKp strain markers. The median age of all cases was 54 years (25th and 75th percentiles, 50.5 to 70), 62.96% of individuals had diabetes, and 22.22% of isolates were sourced from individuals without underlying disease. The ratios of white blood cells/procalcitonin and C-reactive protein/procalcitonin were potential clinical markers for the identification of suppurative infection caused by hvKp and cKp. The 54 K. pneumoniae isolates were classified into 8 sequence type 11 (ST11) and 46 non-ST11 strains. ST11 strains carrying multiple drug resistance genes have a multidrug resistance phenotype, while non-ST11 strains carrying only intrinsic resistance genes are generally susceptible to antibiotics. Bactericidal kinetics revealed that hvKp isolates were not easily killed by antimicrobials at susceptible breakpoint concentrations compared with cKp. Given the varied clinical and molecular features and the catastrophic pathogenicity of K. pneumoniae, it is critical to determine the characteristics of such isolates for optimal management and effective treatment of K. pneumoniae causing pyogenic infections.

**IMPORTANCE** Klebsiella pneumoniae may cause pyogenic infections, which are potentially life-threatening and bring great challenges for clinical management. However, the clinical and molecular characteristics of K. pneumoniae are poorly understood, and effective antibacterial treatment strategies are limited. We analyzed the clinical and molecular features of 54 isolates from patients with various pyogenic infections. We found that most patients with pyogenic infections had underlying diseases, such as diabetes. The ratio of white blood cells to procalcitonin and the ratio of C-reactive protein to procalcitonin were potential clinical markers for differentiating hypervirulent K. pneumoniae strains from classical K. pneumoniae strains that cause pyogenic infections. K. pneumoniae isolates of ST11 were generally more resistant to antibiotics than non-ST11 isolates. Most importantly, hypervirulent K. pneumoniae strains were more tolerant to antibiotics than classic K. pneumoniae isolates.

## INTRODUCTION

Klebsiella pneumoniae remains the most common nosocomial pathogen worldwide, primarily causing infection in patients with underlying disease or immunodeficiency and hospitalized patients (1, 2). In recent years, serious infections caused by these bacteria in healthy populations have been well documented, mainly resulting in liver abscesses, lung abscesses, and endophthalmitis, which are often accompanied by invasive bloodstream infections (2–4). Previous analyses of the bacteria that cause community-acquired liver abscesses revealed that liver abscesses are often caused by hypervirulent K. pneumoniae (hvKp) (5, 6). Subsequently, the clinical and molecular characteristics of K. pneumoniae causing pyogenic liver abscesses were studied (1, 3). Supported by high-throughput sequencing techniques, increasing evidence indicates that the dominating capsular genotypes are the K1 and K2 locus in the molecular epidemiology of hvKp (2, 7). Notably, sequence type 23 (ST23) generally cooccurs with K1 and ST65 overlaps with K2, both of which are the most common types in hvKp (2, 8).

Successfully and accurately defining hvKp remains challenging. In earlier years, the string test was applied to identify hvKp, which is a rapid method, but the accuracy is poor (2, 9). Subsequently, an infection model of Galleria mellonella larvae was used to distinguish hvKp from classical K. pneumoniae (cKp). Based on a better understanding of the above studies, many researchers believe that using a survival-based larval infection model to identify hvKp is not entirely reliable (10). Although the murine infection model is considered to be more reliable in the identification of hvKp, many researchers prefer to score virulence based on the presence of certain genes associated with hvKp. This is mainly because determining the possession of four of five genes that occur on the hvKp-specific virulence plasmid is highly precise in distinguishing hvKp (9–11).

Although data from the available literature have indicated that hvKp is susceptible to widely used antimicrobial drugs, the treatment of patients with severe infections caused by hvKp remains challenging (7, 12). According to several studies, quinolones have some benefits in combating hvKp, but there is still a need to improve the prognosis or enhance the efficacy of antimicrobial strategies (12). Furthermore, resistance to antimicrobial agents in K. pneumoniae is increasing (13). Widespread hypervirulent and multidrug-resistant (MDR) K. pneumoniae isolates pose a serious threat to public health. Therefore, clinical treatment can be supported by determining the most effective antimicrobial treatment strategies and exploring the molecular and clinical characteristics of K. pneumoniae causing pyogenic infection, not just liver abscesses. In this work, we not only reveal the molecular and clinical characteristics of K. pneumoniae causing pyogenic infection but also suggest the bactericidal kinetic effects of important antimicrobial agents on hvKp, providing comprehensive information for the clinical treatment of hvKp.

## RESULTS

### Clinical characteristics.

A total of 54 cases of K. pneumoniae causing pyogenic infection that met the inclusion criteria were included, comprising 28 male patients and 26 female patients, whose median age was 54 years (25th and 75th percentiles, 24 to 87 years). The clinical characteristics are shown in Table 1. A total of 21 liver abscesses, 11 gallbladder abscesses, 8 skin or soft tissue abscesses, 7 renal abscesses, 5 abdominal or pelvic abscesses, and 2 lung abscesses were collected in 54 cases of K. pneumoniae causing pyogenic infection. There were 12 cases (22.22%) without underlying diseases and 42 cases (77.78%) with underlying or concomitant diseases, including 34 cases (62.96%) of diabetes mellitus, 13 cases (24.07%) of hypertension, 12 cases (22.22%) of coronary artery disease, 3 cases (5.56%) of malignancy, 11 cases (20.37%) of hepatitis cirrhosis, 18 cases (33.33%) of gallstones or obstruction, and 15 cases (27.78%) of stones or urinary obstruction. The most common clinical symptoms were fever (81.48%), bloodstream infection (59.26%), which included sepsis (31.48%), and pyogenic shock (22.22%). The median duration of hospitalization was 16 (9.50 to 27.00) days. In addition, significant inflammatory biomarker abnormalities were observed, including increased white blood cell (WBC) count (72.22%), elevated C-reactive protein (CRP) levels (88.89%), and elevated procalcitonin (PCT) levels (70.37%). After treatment, 75.93% of the patients had a good prognosis.

**Table 1:** Clinical characteristics of K. pneumoniae causing pyogenic infections^*a*^

Clinical characteristic	K. pneumoniae (*n* = 54)	hvKp (*n* = 33)	cKp (*n* = 21)	*P* value
Age, years	54.00 (24.00–87.00)	60.50 (24.00–87.00)	64.00 (26.00–86.00)	0.374
Gender				
Male	28.00 (51.85)	16.00 (48.48)	12.00 (57.14)	0.534
Female	26.00 (48.15)	17.00 (51.52)	9.00 (42.86)	
Underlying or concomitant conditions				
Diabetes mellitus	34.00 (62.96)	21.00 (63.64)	11.00 (52.38)	1.000
Hypertension	13.00 (24.07)	8.00 (24.24)	5.00 (23.81)	1.000
Coronary heart disease	12.00 (22.22)	6.00 (18.18)	6.00 (28.57)	0.329
Malignancy	3.00 (5.56)	1.00 (3.03)	2.00 (9.52)	0.555
Hepatitis or cirrhosis	11.00 (20.37)	3.00 (9.09)	8.00 (38.1)	0.017
Gallstones or obstruction	18.00 (33.33)	10.00 (30.3)	8.00 (38.1)	0.389
Calculus or obstruction of urinary system	15.00 (27.78)	11.00 (33.33)	4.00 (19.05)	0.938
No underlying diseases	12.00 (22.22)	7.00 (21.21)	5.00 (23.81)	1.000
Clinical symptoms				
Fever	44.00 (81.48)	26.00 (78.79)	18.00 (85.71)	1.000
Bloodstream infection	32.00 (59.26)	17.00 (51.52)	15.00 (71.439)	0.183
Septicemia	17.00 (31.48)	9.00 (27.27)	8.00 (38.1)	0.447
Septic shock	12.00 (22.22)	7.00 (21.21)	5.00 (23.81)	1.000
Laboratory examination				
WBC	12.78 (9.59–18.72)	14.71 (10.26–18.74)	16.92 (8.35–19.10)	0.801
PLT	153.00 (93.5–252.0)	183.90 (102.00–278.00)	139.00 (73.50–200.5)	0.172
CRP	138.85 (64.20–189.30)	142.30 (91.45–216.75)	98.05 (35.82–184.75)	0.162
PCT	12.17 (0.78–93.57)	4.23 (0.60–73.20)	24.80 (4.14–125.88)	0.056
CRP/PCT	9.81 (1.52–84.83)	19.24 (2.78–109.81)	3.52 (0.77–40.00)	0.017
WBC/PCT	1.53 (0.22–11.59)	3.41 (0.27–14.24)	0.87 (0.14–4.14)	0.042
Disease				
Liver abscess	21.00 (38.89)	15.00 (45.45)	6.00 (28.57)	0.215
Gallbladder abscess	11.00 (20.37)	4.00 (12.12)	7.00 (33.33)	0.085
Skin or soft tissue abscess	8.00 (14.81)	6.00 (18.18)	2.00 (9.52)	0.461
Abdominal or pelvic abscess	5.00 (9.25)	3.00 (9.09)	2.00 (9.52)	1.000
Renal abscess	7.00 (12.96)	4.00 (12.21)	3.00 (18.29)	1.000
Lung abscess	2.00 (3.70)	1.00 (3.03)	1.00 (4.76)	1.000
Length of stay in hospital, days^*b*^	16.00 (9.50–27.00)	18.00 (12.00–27.00)	13.00 (8.50–25.00)	0.392
Admission to ICU	22.00 (40.74)	11.00 (33.33)	11.00 (52.38)	0.164
Expenditure	62,842.11 ± 163,645.20	28,444.70 ± 13,990.31	116,158.08 ± 255,423.81	0.002
Prognosis				
Good	41.00 (75.93)	26.00 (78.79)	15.00 (71.439)	0.507
Poor or withdrawal from treatment	12.00 (22.22)	6.00 (18.18)	6.00 (28.57)	

a Values are presented as medians (with 25th and 75th percentiles), means ± standard deviations, or number (and percent) of patients. WBC, white blood cell; PLT, platelet; CRP, C-reactive protein; PCT, procalcitonin; ICU, intensive care unit; hvKp, hypervirulent Klebsiella pneumoniae; cKp, classical Klebsiella pneumoniae.

b Length of stay was >3 days.

**FIG 1:**
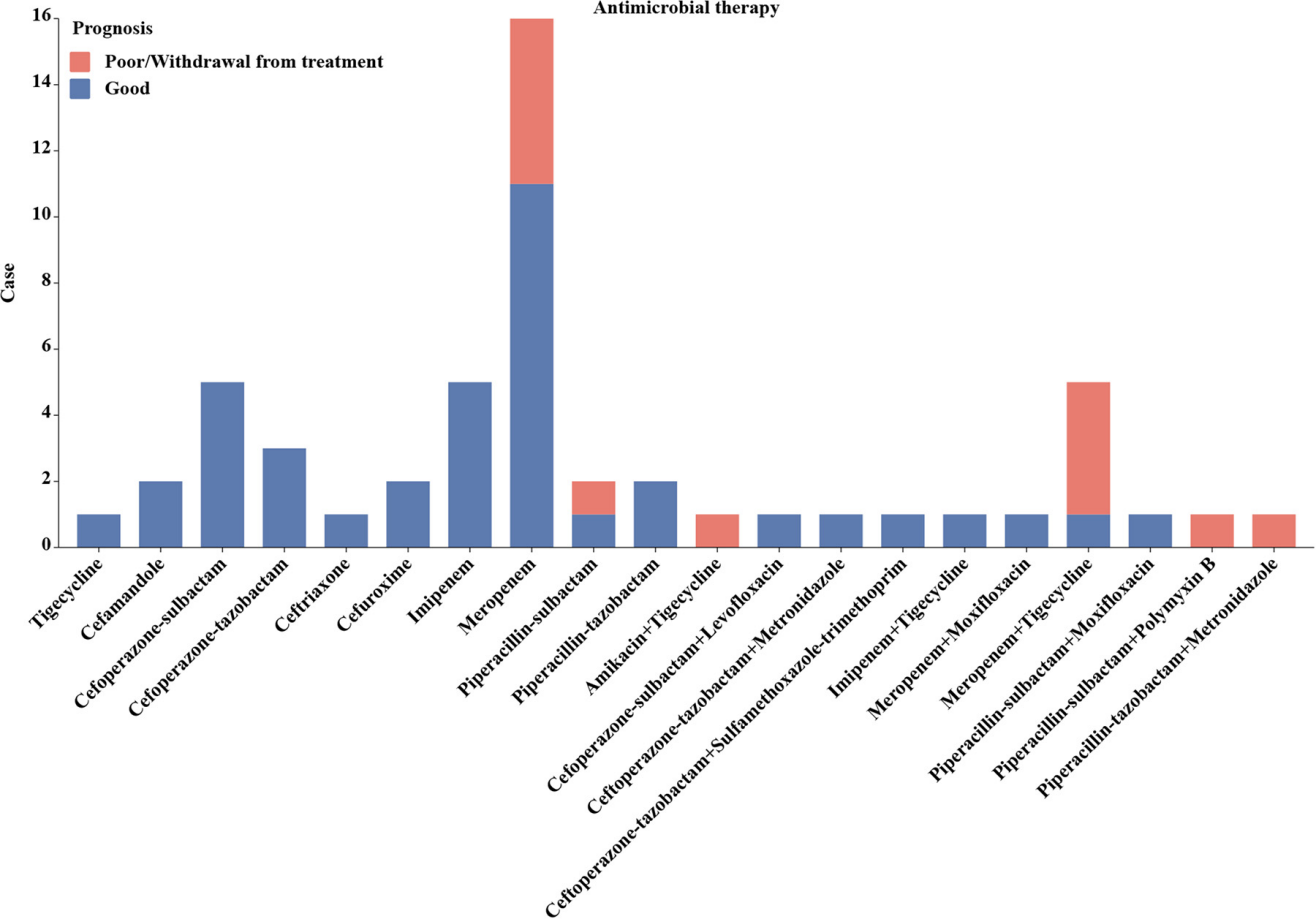
Antimicrobial therapy classification and prognosis of included cases treated with antimicrobial agents.

Out of 54 patients, 53 received antimicrobial therapy (Fig. 1). Among these patients, four were discharged before complete control of the disease. Monotherapy was administered to 39 patients, of whom 7 had a poor prognosis (17.9%). Meropenem was the most commonly used antimicrobial agent for monotherapy in this hospital, and it was effective in 11 of 16 patients (68.75%). Fourteen patients received multidrug combination therapy, of whom 7 had a poor prognosis. Among these seven patients, four were diagnosed with septic shock upon admission, and one patient was under immunosuppression therapy after kidney transplantation.

### Identification of hvKp and cKp.

We identified hvKp by checking for the presence of four of five virulence genes (*iroB*, *iucA*, *rmpA*, *rmpA2*, and *peg-344*). Among the 54 K. pneumoniae isolates, 33 (61.11%) were identified as hvKp, while 21 (38.89%) were identified as cKp. Out of the 33 hvKp isolates, 28 had all five virulence genes uniformly present. Four isolates harbored *iucA*, *rmpA*, *rmpA2*, and *peg-344*, but *iroB* was not found. One isolate, kp398, harbored *iroB*, *iucA*, *rmpA*, and *peg-344*, but *rmpA2* was not detected (see Table S1 in the supplemental material). We also used the string test to identify hvKp isolates and found that the sensitivity, specificity, and positive and negative predictive values of the string test for identifying hvKp were 75.75%, 90.47%, 92.59%, and 70.37%, respectively.

### Comparison of clinical characteristics of hvKp and cKp.

A total of 33 patients with hvKp infection were included in this study, while the remaining 21 patients had cKp infection. Table 1 summarizes the differences in the characteristics of these two groups. The hvKp group had a median age of 60.5 years (24 to 87), and the cKp group had a median age of 64 years (26 to 86), with no significant difference (*P* = 0.374). The hvKp group had a lower prevalence of hepatitis or cirrhosis than the cKp group (9.09% versus 38.10%; *P* = 0.017). Interestingly, there was no significant difference in the results when WBC, PCT, and CRP were compared separately (*P* > 0.05), but the WBC/PCT ratio (*P* = 0.042) and the CRP/PCT ratio (*P* = 0.017) were lower in the cKp group than in the hvKp group.

### Antimicrobial susceptibility of K. pneumoniae causing pyogenic infections.

We analyzed the susceptibility of 54 K. pneumoniae strains to clinically available first-line antimicrobials (Table 2). The results demonstrated that the resistance rate toward commonly used antibiotics was below 30%, except for ampicillin, whose resistance is conferred intrinsically by chromosomally encoded beta-lactamases. Importantly, the antimicrobial resistance of ST11 types nearly represented the resistance for all K. pneumoniae strains, as ST11 exhibited MDR, whereas non-ST11 types showed the lowest resistance to the listed antimicrobials (Table 3). In addition, we compared the susceptibility of hvKp and cKp to the listed antimicrobials (Table 2). Surprisingly, there was no significant difference in resistance levels between the two groups (*P* > 0.05), except for sulfamethoxazole-trimethoprim, suggesting that the distribution of MDR phenotypes is balanced among hvKp and cKp causing pyogenic infections (Table 2).

**Table 2:** Susceptibility of K. pneumoniae to popular antimicrobial agents

Antimicrobial agent	% of isolates resistant or susceptible^*a*^						*P* value
	K. pneumoniae		cKp		hvKp		
	(*n* = 54)		(*n* = 22)		(*n* = 33)		
	R	S	R	S	R	S	
Amikacin	14.8	85.2	14.3	85.7	15.2	84.8	0.626
Gentamicin	16.7	83.3	19.0	81.0	15.2	84.8	0.429
Cefoperazone-sulbactam	14.8	85.2	14.3	85.7	15.2	84.8	0.626
Piperacillin-tazobactam	16.7	83.3	19.0	81.0	15.2	84.8	0.429
Imipenem	14.8	85.2	14.3	85.7	15.2	84.8	0.626
Meropenem	14.8	85.2	14.3	85.7	15.2	84.8	0.626
Cefazolin	29.6	70.4	38.1	61.9	24.2	75.8	0.217
Cefuroxime	25.9	72.2	38.1	61.9	18.2	78.8	0.202
Ceftazidime	25.9	74.1	38.1	61.9	18.2	81.8	0.123
Ceftriaxone	25.9	74.1	38.1	61.9	18.2	81.8	0.123
Cefepime	22.2	77.8	33.3	66.7	15.2	84.8	0.110
Cefoxitin	18.5	81.5	23.8	76.2	15.2	84.8	0.326
Ciprofloxacin	24.1	64.8	38.1	61.9	15.2	75.8	0.109
Levofloxacin	22.2	63.0	33.3	47.6	15.2	72.7	0.077
Sulfamethoxazole-trimethoprim	24.1	75.9	57.1	42.9	3.0	97.0	0.000
Ampicillin	100.0	0	100.0	0	100.0	0	1.000

a Abbreviations: R, resistant; S, susceptible; hvKp, hypervirulent Klebsiella pneumoniae; cKp, classical Klebsiella pneumoniae.

**Table 3:** Susceptibility of ST11 and non-ST11 K. pneumoniae to popular antimicrobial agents

Antimicrobial agent	% of isolates resistant or susceptible^*a*^			
	ST11 (*n* = 8)		Non-ST11 (*n* = 46)	
	R	S	R	S
Amikacin	87.5	12.5	2.2	97.8
Gentamicin	87.5	12.5	4.4	95.7
Cefoperazone-sulbactam	100.0	0	2.2	97.8
Piperacillin-tazobactam	100.0	0	4.4	95.7
Imipenem	100.0	0	0	100.0
Meropenem	100.0	0	0	100.0
Cefazolin	100.0	0	17.4	82.6
Cefuroxime	100.0	0	15.2	84.8
Ceftazidime	100.0	0	13.0	87.0
Ceftriaxone	100.0	0	13.0	87.0
Cefepime	100.0	0	8.7	91.3
Cefoxitin	100.0	0	4.4	95.7
Ciprofloxacin	100.0	0	8.7	91.3
Levofloxacin	100.0	0	8.7	91.3
Sulfamethoxazole-trimethoprim	50.0	50.0	19.6	80.4
Ampicillin	100.0	0	100	0

a Abbreviations: R, resistant; S, susceptible.

### Clinical and molecular characteristics of K. pneumoniae.

**FIG 2:**
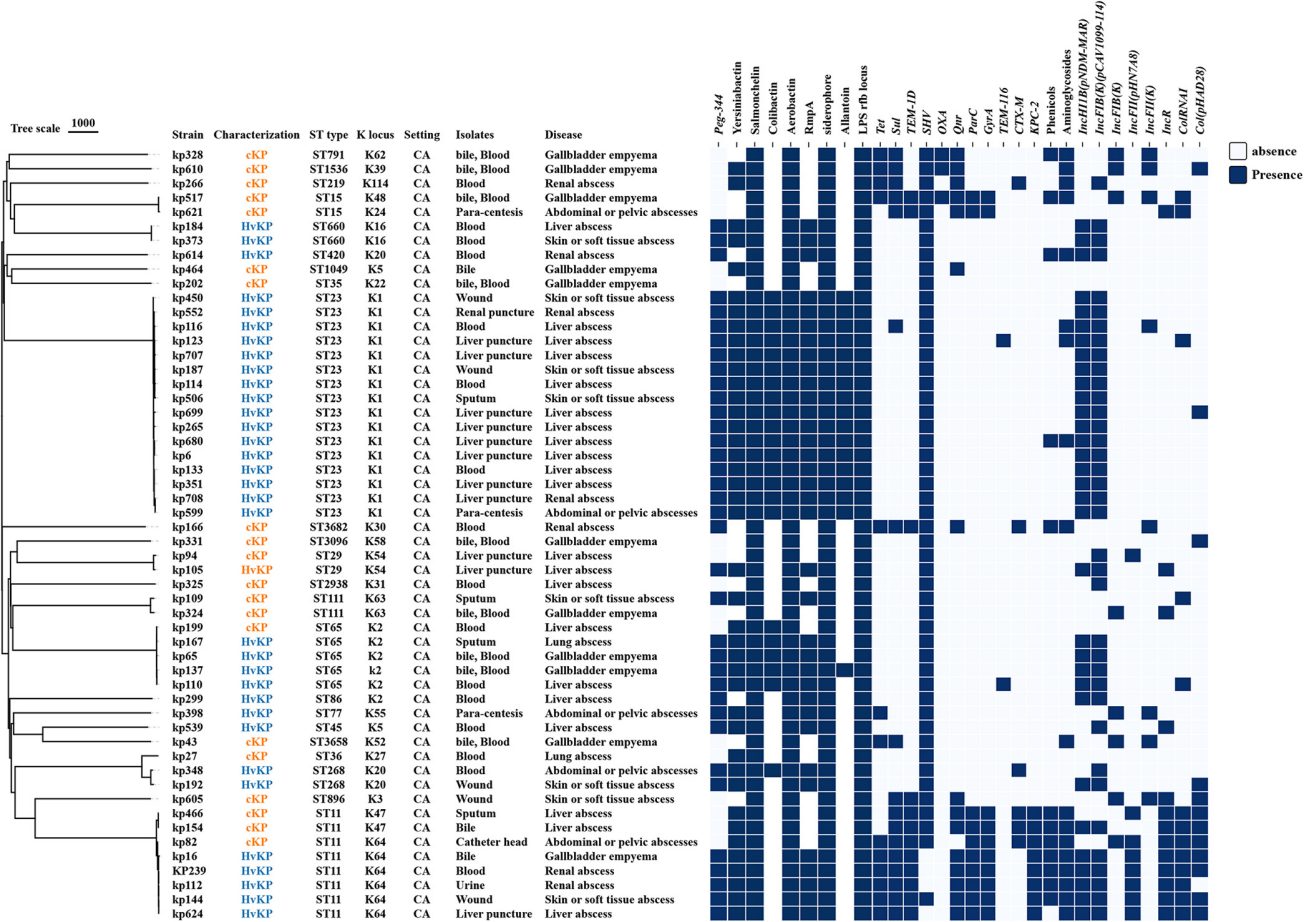
Phylogenetic tree, source of isolates, diseases, strains, and molecular characterization of 54 *Klebsiella pneumoniae* causing pyogenic infections. Strain names, strain characteristics, ST, K locus, source of isolates, diseases, virulence factors, antimicrobial resistance genes, and plasmid replicon are shown from left to right. CA, community acquired; hvKp, hypervirulent Klebsiella pneumoniae; and cKp, classical Klebsiella pneumoniae.

To determine the molecular characteristics of KP-FP, whole-genome sequencing was performed. According to multilocus sequence typing and nucleotide sequences of capsule synthesis locus type (K locus), the 54 K. pneumoniae strains were classified into 23 ST types and 22 K locus types, which comprised 16 ST23 strains, 8 ST11 strains, and 30 strains with other ST types. The most frequently observed K type was K1 (29.6%, 16/54), followed by K2 (11.1%, 6/54) and K64 (11.1%, 6/54). As expected, all ST23 types were paired with the K1 serotype, while ST11 was paired with K64 and K47 (Fig. 2). Consistent with previous findings, the ST11 type exhibited a higher prevalence of antibiotic resistance genes than non-ST11 types (14).

Furthermore, we analyzed the genes encoding virulence factors that were associated with invasive infection, including the regulator of mucoid phenotype A (*rmpACD* and/or *rmpA2*), aerobactin, yersiniabactin, salmochelin (*iro*), colibactin (*clb*), enterobactin, lipopolysaccharide (*LPS*), AcrAB, allantoin (*all*), kfuABC, rcsAB (*rsc*), fimbriae, and type VI secretion system (T6SS)-I, T6SS-II, and T6SS-III genes (Fig. 2 and Supplementary Fig. S1). We found that 100% (54/54) of strains contained virulence genes associated with salmochelin, lipopolysaccharide, siderophore, and aerobactin; 75.93% (41/54) of the isolates carried yersiniabactin-related virulence genes; 40.74% carried colibactin-related virulence genes; 64.81% carried the *rmpA* virulence gene; and 31.48% of isolates carried allantoin-related virulence genes. A phylogenetic tree based on single-nucleotide polymorphisms revealed that the strains could be classified into multiple clusters, which were generally in accordance with their STs (Fig. 2). Notably, strains of hvKp and cKp with identical STs were grouped, implying that hvKp and cKp are not evolutionarily distinct, which is consistent with previous reports (15, 16).

Given the critical significance of resistance genes for clinical purposes, we profiled the resistance genes of K. pneumoniae isolates on the BacWGSTdb website. Except for ST11, which carries MDR genes, a majority of non-ST11 strains carried only a few intrinsic resistance genes (see Fig. S2 in the supplemental material and Fig. 2), which was consistent with the results of antimicrobial susceptibility testing (AST) (Table 2).

### Comparison of molecular characteristics of hvKp and cKp.

**FIG 3:**
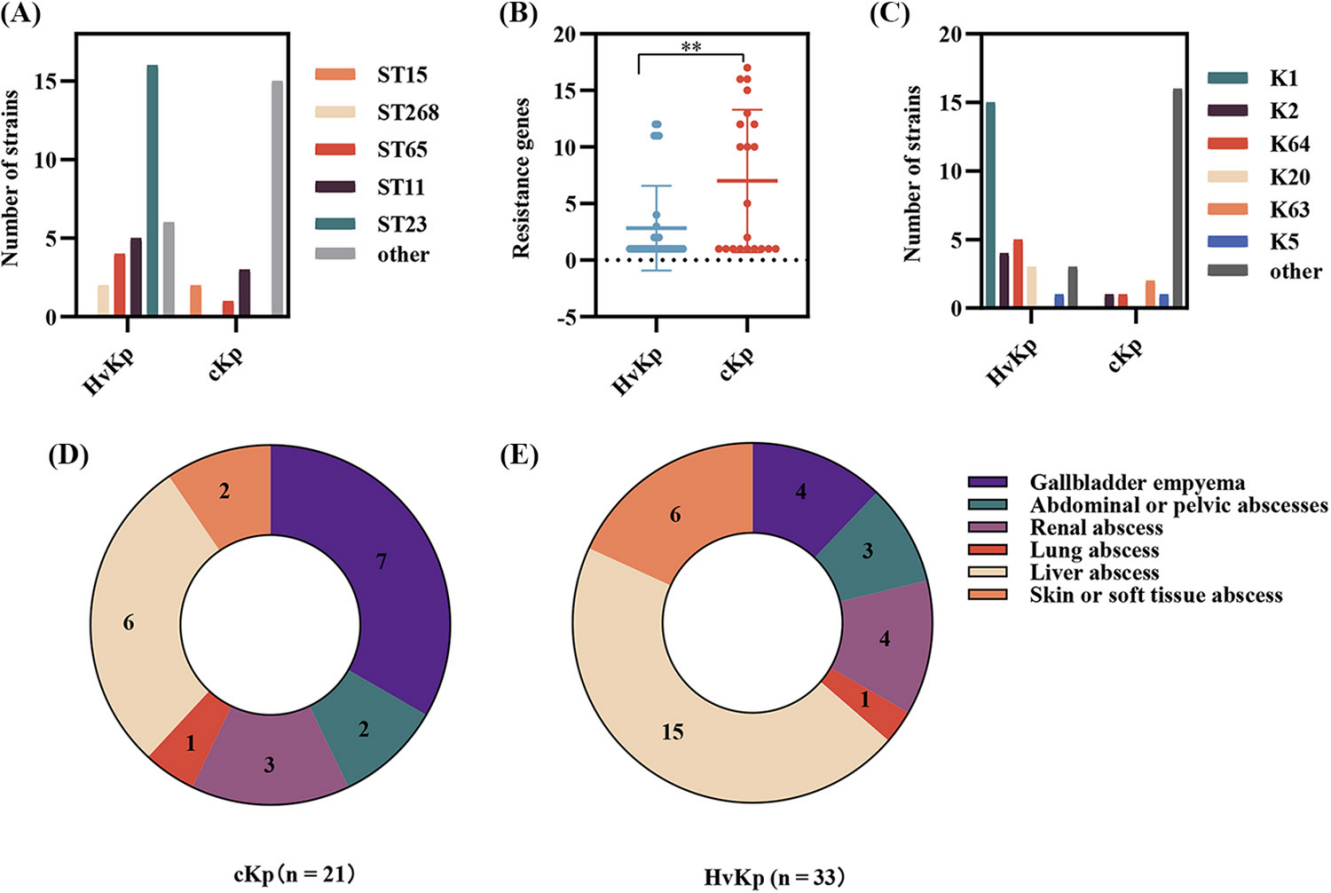
Comparison of the molecular characteristics of hvKp and cKp. (A) Distribution of different STs between hvKp and cKp. (B) Comparison of the number of resistance genes between hvKp and cKp. (C) Distribution of different K loci between hvKp and cKp. (D and E) Distribution of infected foci of cKp (D) and hvKp (E). hvKp, hypervirulent Klebsiella pneumoniae; cKp, classical Klebsiella pneumoniae. **, *P* < 0.01.

To understand the differences between hvKp and cKp, we compared the MLST, K locus, and resistance genes of these strains. The results showed that the ST23 type was dominant in hvKp and paired with K1, but the MLST and K locus distribution in cKp exhibited diversity. Interestingly, although the number of resistance genes was lower in hvKp than in cKp, there was no difference in the resistance levels between them, which may be because the genes conferring resistance to several classes of antibiotics were evenly distributed in K. pneumoniae causing pyogenic infection (Fig. 3A to C and Fig. 2). In terms of the site of pyogenic infection, both hvKp and cKp can cause multisite pyogenic infection. We found that 15 cases of pyogenic liver infection and 6 cases of pyogenic skin or soft tissue infection were caused by hvKp, while 6 cases of pyogenic liver infection and 7 cases of pyogenic gallbladder infection were caused by cKp (Fig. 3D and E).

### Bactericidal kinetic effects of critical antimicrobial drugs against highly virulent K. pneumoniae.

**FIG 4:**
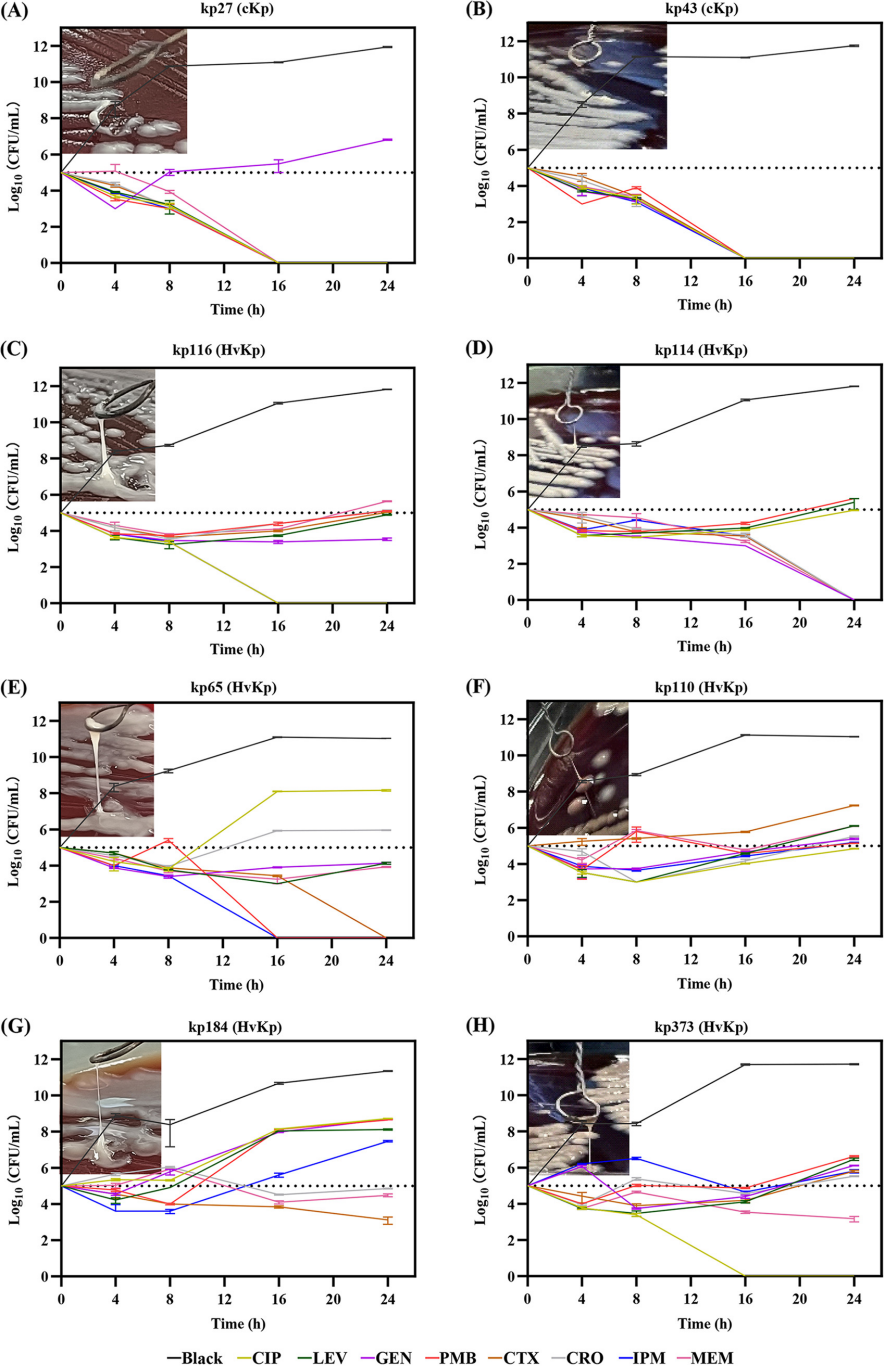
*In vitro* time-kill assayresults using average steady-state serum concentrations of ciprofloxacin (CIP), levofloxacin (LEV), polymyxin (PMB), gentamicin (GEN), ceftriaxone (CRO), cefotaxime (CTX), meropenem (MEM), and imipenem (IPM) against K. pneumoniae isolates. (A and B) Two classical K. pneumoniae isolates with viscous strings of <5 mm. (C and D) Two K1-ST23 hypervirulent K. pneumoniae isolates with viscous strings of >5 mm. (E and F) Two K2-ST65 hvKp isolates with viscous strings of >5 mm. (G and H) Two K16-ST660 hvKp isolates with viscous strings of >5 mm.

Rapid killing of highly virulent K. pneumoniae is a major challenge for clinicians to treat patients with severe infections. Therefore, we performed time-kill assays to analyze the efficiency of antimicrobials against strains with different characteristics. Our findings showed that all antimicrobials except for gentamicin had a higher minimum bactericidal concentration value for hvKp than for cKp (see Table S2). It was also confirmed through the time-kill assays that it is difficult to kill hvKp rapidly with highly effective antimicrobials (Fig. 4). Furthermore, we observed that the broad-spectrum antimicrobials, including imipenem, meropenem, and polymyxin B, had no obvious advantage in killing hvKp (Fig. 4). Of course, it was also necessary that the antimicrobial of long-term and high-dose were applied to treat *Klebsiella pneumoniae* causing pyogenic infections.

## DISCUSSION

K. pneumoniae infection is a serious public health threat due to the increasing prevalence of infections caused by MDR K. pneumoniae worldwide (17, 18). According to the most recent data from the China Antimicrobial Surveillance Network (13), K. pneumoniae accounted for 13.99% of all infections in a collection of 339,513 bacterial isolates from China in 2022. K. pneumoniae can cause pyogenic infections, such as pyogenic liver abscesses, skin, and soft tissue infections, including necrotizing fasciitis (19, 20), abdominal and pelvic abscesses (2), renal abscesses (21), gallbladder abscesses (22), and lung abscesses (2, 3). However, there is limited understanding regarding the molecular characteristics of this bacterium, the clinical features of the pyogenic infections it causes, and the molecular-clinical associations involved. To address the above questions, we explored the clinical and genetic characteristics of K. pneumoniae isolates obtained in this study.

Previous studies have described diabetes mellitus as a risk factor for pyogenic liver abscesses (23, 24). In the present study, our data showed that the incidence of diabetes among patients with pyogenic infection was as high as 62.96% (34/54). In addition, long-term high blood glucose levels can damage various organs and systems, leading to a range of health problems (25). Poorly controlled blood glucose can impair the phagocytic function of neutrophils, affect the absorption and utilization of antimicrobials, and promote the growth of pathogens in tissues, and metabolic disorders may have negative effects on organs (26, 27). In the present study, out of 12 patients without any preexisting health conditions, seven (58.33%) had hvKp infection, implying that hvKp has the potential to cause infections in otherwise-healthy individuals (28). Patients with hepatitis or cirrhosis were more likely to be infected with cKp in the present study. cKp is more resistant or recurring, resulting in higher costs (26). Moreover, the majority of patients exhibit signs of inflammation and fever, and our findings showed that the ratios of WBC/PCT and CRP/PCT were potential clinical markers for identifying suppurative infection caused by hvKp and cKp, which is consistent with previous reports showing that CRP and PCT are reliable indicators to differentiate between hvKp and cKp (29, 30).

Based on the presence of specific marker genes (9, 11), the majority (33 of the 54 cases) of pyogenic infections were caused by hvKp, which is consistent with its characteristics of being more virulent and aggressive (2, 3). The results of this study indicated that K1-ST23 types were the most prevalent hvKp clones and that these were primarily observed in liver abscesses, in agreement with reports from China and other Asian countries (31, 32). Additionally, numerous other capsular-sequence type combinations were identified, which included well-established K2-ST86 type hvKp isolates, as well as newly discovered isolates with K55-ST77. Small differences may exist due to virulence gene patterns caused by insertions and deletions, which are known to occur in pLVPK-related virulence plasmids (33, 34). It has been observed that strains causing liver abscesses possess numerous virulence genes that correspond to the hypervirulent phenotype (15). More than half of the isolates in this investigation included 11 virulence factors, especially in liver abscesses, and the longest hospital stay was 137 days, which may reflect the resistant character of pyogenic K. pneumoniae infections. This could be due to the small size of our sample, which could result in some bias. Biomarkers and string tests can be used to identify hvKp, which can aid in the diagnosis of pyogenic infections and antibiotic therapy. Although string testing is a quick method for identifying hvKp in clinical microbiology laboratories, this study and others have demonstrated that it is insufficiently sensitive and specific to detect hvKp alone (9, 10).

As its resistance to the majority of first-line antibiotics grows, hvKp is rapidly receiving attention. We compiled the resistance gene profiles of K. pneumoniae from the BacWGSTdb database and found that all the strains included *bla*_SHV_, as reported previously (4, 33, 35, 36). Interestingly, only the ST11 type exhibited significant resistance in the hvKp population of this study, while all other hvKp strains were found to not carry additional resistance genes, displaying high susceptibility to commonly used antimicrobial agents. The initially reported hvKp strains were susceptible, but over time, they may have gradually acquired resistance through mutations in resistance genes and the transfer of resistance plasmids (37, 38). The bactericidal properties of antimicrobial agents against hvKp were revealed by time-kill assays, which indicated that hvKp is not easily killed by antimicrobials at sensitive breakpoint concentrations compared with cKp, and that imipenem, meropenem, and polymyxin B have no significant killing effect. We speculate that the high tolerance of hvKp to antimicrobials may be associated with a hypermucoviscous phenotype. The increased virulence of hvKp may lead to the formation of biofilms, antibiotic heteroresistance, or toxin-antitoxin systems (39–41). Therefore, prolonged therapy with high doses of antimicrobials may be required to treat K. pneumoniae causing pyogenic infections.

Although we tried to reveal the clinical and molecular characteristics of *Klebsiella pneumoniae* causing pyogenic infections and support antibacterial treatment strategies from multiple dimensions through some well-designed and rigorous clinical case screening, there are several limitations to our study. First, the number of strains included is small, which makes the generalization of certain observed differences in clinical and molecular characteristics between cKp and hvKp relatively weak. Moreover, this study was conducted in only one hospital, and hvKp infections in other countries may have different clinical characteristics, especially if distinct hvKp clones are involved.

In conclusion, we investigated the clinical and molecular characteristics of K. pneumoniae strains isolated from patients with pyogenic infections, which led to a more thorough understanding and better management of K. pneumoniae-caused pyogenic infections. Notably, the WBC/PCT and CRP/PCT ratios may be effective clinical indicators to distinguish between hvKp and cKp pyogenic infections. *In vitro* time-kill assays showed that hvKp was more difficult to rapidly kill than cKp. Furthermore, ST11 K. pneumoniae associated with two K loci (K47 and K64) may acquire the carbapenem-hydrolyzing *bla*_KPC-2_ gene and virulence genes through horizontal gene transfer. Therefore, hospitals must implement stringent infection control measures to prevent the spread of carbapenem-resistant K. pneumoniae.

## MATERIALS AND METHODS

### Inclusion criteria and clinical data collection.

Patients with K. pneumoniae infection who were admitted to our hospital between 2018 and 2021 were considered for inclusion. Inclusion and exclusion criteria were based on clinical symptoms, physical exams, laboratory markers, imaging, and clinical diagnosis. Inclusion criteria were as follows: (i) a clear pyogenic infection site was confirmed by physical examination and/or imaging diagnosis; (ii) K. pneumoniae was isolated from the infection specimen; and (iii) the respiratory tract purulent sputum specimen was identified as a qualified specimen, with the number of white blood cells being greater than 25 and the number of epithelial cells being fewer than 10 at low magnification. Exclusion criteria were as follows: (i) pyogenic infections not caused by K. pneumoniae; (ii) K. pneumoniae was isolated from unqualified pyogenic infection specimens. To determine the clinical characteristics of K. pneumoniae causing pyogenic infection, clinical diagnosis and treatment data were extracted from electronic medical records, including specimen source and patient characteristics, such as age, gender, underlying diseases, clinical symptoms, imaging and laboratory data, treatment procedures, prognosis, and outcome.

### Bacterial strains.

In total, 54 nonduplicate clinical K. pneumoniae isolates were obtained from inpatients at The Second Affiliated Hospital, Hengyang Medical School, University of South China, Hunan, China, from 2018 to 2021. Strains were isolated from blood, wound pus, puncture fluid, pyuria, or purulent sputum from pyogenic infection inpatients and identified as K. pneumoniae using a Microbial ID/AST system DL-96II (DL Biotechnology Co., Ltd., Zhuhai, China) and 16S rRNA gene sequencing.

### Antimicrobial susceptibility testing.

The MICs of ampicillin, aztreonam, cefoxitin, cefazolin, cefuroxime, ceftriaxone, ceftazidime, cefepime, ampicillin-sulbactam, piperacillin-tazobactam, imipenem, meropenem, ciprofloxacin, levofloxacin, gentamicin, amikacin, and sulfamethoxazole-trimethoprim were determined using the broth microdilution method. The AST results were interpreted based on the latest M100-ED32 guidelines of the Clinical and Laboratory Standards Institute (CLSI). The Escherichia coli reference strain ATCC 25922 was used as quality control for AST. MDR was defined as resistance to at least one antimicrobial agent in three or more antimicrobial categories (42, 43).

### String test assays.

The string test was performed as described previously (2). An inoculation loop was used to stretch K. pneumoniae grown on a blood agar plate, and the formation of viscous strings of >5 mm in length was used to define the hypermucoviscous phenotype. A positive string test also suggested that the bacteria had hypervirulent characteristics.

### Whole-genome sequencing and bioinformatics analysis.

All strains were inoculated on Columbia blood agar plates and incubated at 37°C overnight. The purified colony was cultured in 3 mL of LB broth at 37°C for 12 to 14 h. Genomic DNA was extracted from bacteria using a kit (TaKaRa, Dalian, China). The NEBNext Ultra II DNA library preparation kit was used to prepare a 400-bp library for Illumina Novaseq high-throughput sequencing. Raw reads were filtered with Trimmomatic and evaluated using FastaQC. Cleaned reads were assembled using Spades (44). Scaffold sequences were annotated using the RAST annotation server (45). Kaptive software was used to analyze capsular serotypes (K locus) (46). Multilocus sequence typing and ARG analysis were performed using the BacWGSTdb webserver (http://bacdb.cn/BacWGSTdb/) (47). A neighbor-joining tree based on core genome single-nucleotide polymorphisms was constructed within the Pathogenwatch platform, which comprises a core gene set of 1,972 genes (48).

### Identification of virulence genes and hvKp.

The Spades-assembled draft genome sequences of the 54 K. pneumoniae isolates were uploaded to the VFanalyzer online tool of the Virulence Factors Database to search for virulence factors. The VFanalyzer pipeline integrates orthologous groups construction, iterative sequence similarity searches, and context-based data refinement to identify virulence factors with high specificity and sensitivity (49). The reference strain used in the search process was NTUH-K2044. Adherence, biofilm formation, efflux pumps, immunological evasion, iron uptake, nutritional factors, regulation, secretion systems, serum resistance, and toxin-related genes are Klebsiella's key virulence factors (50). K-PAM (www.iith.ac.in/K-PAM/) can be used to identify hypervirulent Klebsiella spp. from conventional Klebsiella spp. using the marker genes *iroB*, *iucA*, *rmpA*, *rmpA2*, and *peg-344* (11). The server classifies a strain as hvKp if four of the five genes are present (10, 11, 51).

### Time-kill assay.

As previously described (52), time-kill assays were conducted to evaluate the antimicrobial activity of the selected antibiotics for eight K. pneumoniae strains, including two cKp strains (sample numbers kp27 and kp43) and six hvKp strains with positive string test results, namely, K1-ST23-hvKp (kp116 and kp114), K2-ST65-hvKp (kp65 and kp110), and non-K1/K2-hvKp (K184 and K373). These strains possess similar susceptibility to selected antibiotics. The following antibiotic concentrations were used based on the antimicrobial susceptibility breakpoint of CLSI: cefotaxime (1 μg/mL), ceftriaxone (1 μg/mL), imipenem (1 μg/mL), meropenem (1 μg/mL), levofloxacin (0.5 μg/mL), ciprofloxacin (0.25 μg/mL), gentamicin (4 μg/mL), and polymyxin B (2 μg/mL).

### Statistical analysis.

All statistical analyses were performed using SPSS 25.0 software. Categorical variables are expressed as percentages and were analyzed using the chi-square test or Fisher's exact probability method; continuous variables are expressed as means ± standard deviations or as medians (with quartiles), depending on the distribution, and were analyzed using the independent samples *t* test or the Mann-Whitney U test. A *P* value of < 0.05 was considered statistically significant.

### Ethics approval.

This study was designed according to the Declaration of Helsinki (2013) and has been approved by the Ethics Committee in Clinical Research of The Second Affiliated Hospital, University of South China (2022k1201).

### Data availability.

The assembled genome sequences have been deposited at NCBI with the following BioProject ID: PRJNA905661. The data sets generated and/or analyzed during the current study are available from the corresponding author upon reasonable request.
