# Supplementary figures and images for: Clinical and Molecular Characteristics and Antibacterial Strategies of Klebsiella pneumoniae in Pyogenic Infection
Source: Microbiol Spectr. 2023 Jun 21;11(4):e00640-23. doi: 10.1128/spectrum.00640-23 (PMC10434161; doi:10.1128/spectrum.00640-23)

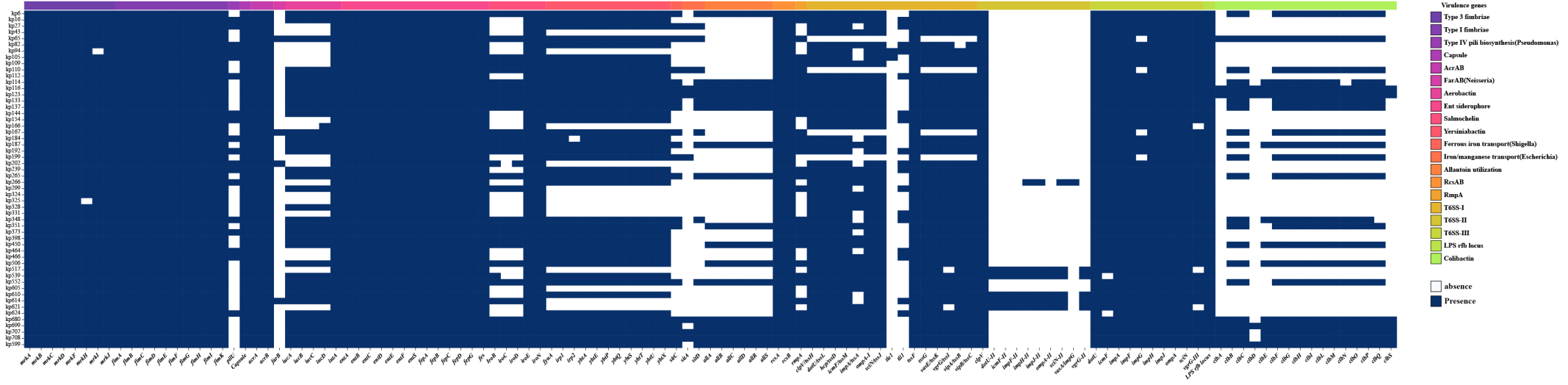

Supplement: Supplemental file 1 — Figure S1. Download spectrum.00640-23-s0001.pdf, PDF file, 1.4 MB [file spectrum.00640-23-s0001.pdf]

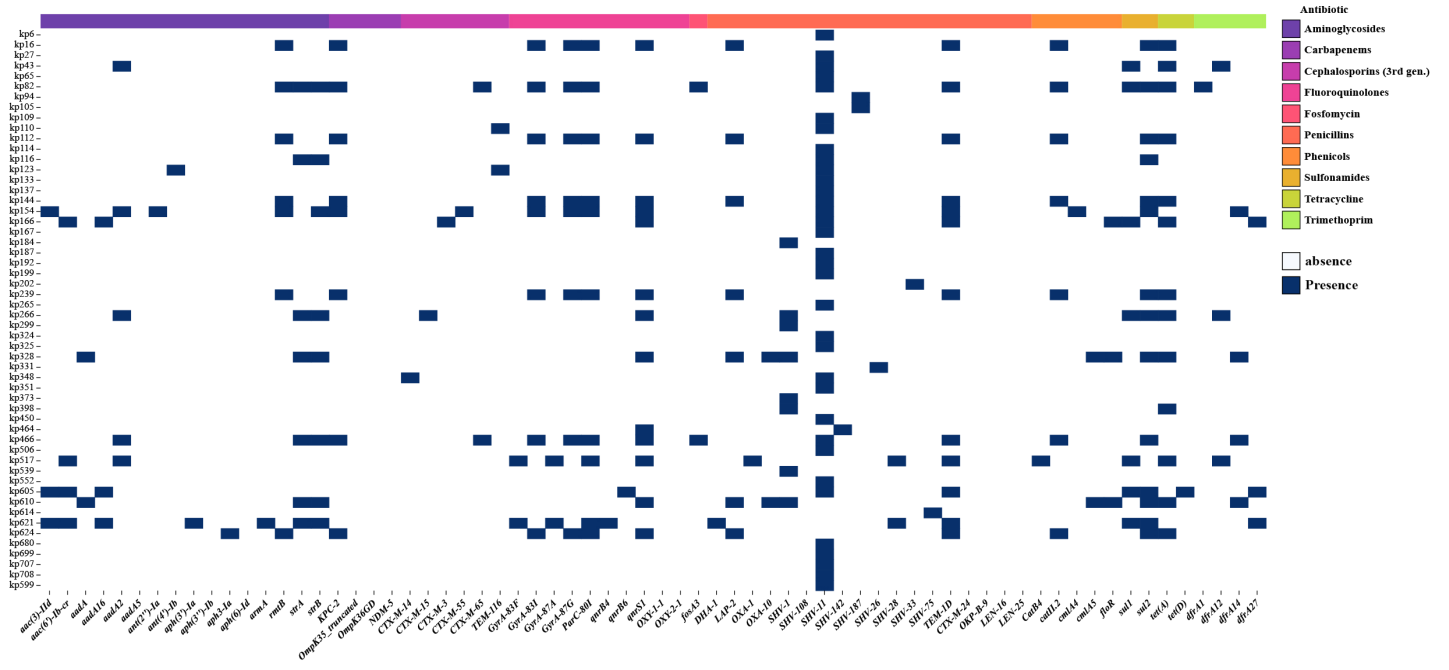

Supplement: Supplemental file 2 — Figure S2. Download spectrum.00640-23-s0002.pdf, PDF file, 0.8 MB [file spectrum.00640-23-s0002.pdf]
