# Supplementary material for: Clinical and Molecular Characteristics and Antibacterial Strategies of Klebsiella pneumoniae in Pyogenic Infection
Source: Microbiol Spectr. 2023 Jun 21;11(4):e00640-23. doi: 10.1128/spectrum.00640-23 (PMC10434161; doi:10.1128/spectrum.00640-23)
Supplement: Supplemental file 3 — Table S1. Download spectrum.00640-23-s0003.docx, DOCX file, 0.03 MB [file spectrum.00640-23-s0003.docx]

Table S1. Identification of HvKp and cKp based on virulence biomarker gene

| Strain  No. | String  test | Virulence geneseb | | | | | Characterizationa |
| --- | --- | --- | --- | --- | --- | --- | --- |
|  |  | *rmpA* | *rmpA2* | *iroB* | *iucA* | *peg-344* |  |
| kp16 | + | + | + | - | + | + | Hypervirulent K. pneumoniae (HvKp) |
| kp112 | - | + | + | - | + | + | Hypervirulent K. pneumoniae (HvKp) |
| kp144 | + | + | + | - | + | + | Hypervirulent K. pneumoniae (HvKp) |
| kp239 | + | + | + | - | + | + | Hypervirulent K. pneumoniae (HvKp) |
| kp624 | + | + | + | + | + | + | Hypervirulent K. pneumoniae (HvKp) |
| kp6 | + | + | + | + | + | + | Hypervirulent K. pneumoniae (HvKp) |
| kp114 | - | + | + | + | + | + | Hypervirulent K. pneumoniae (HvKp) |
| kp116 | + | + | + | + | + | + | Hypervirulent K. pneumoniae (HvKp) |
| kp123 | + | + | + | + | + | + | Hypervirulent K. pneumoniae (HvKp) |
| kp133 | + | + | + | + | + | + | Hypervirulent K. pneumoniae (HvKp) |
| kp187 | + | + | + | + | + | + | Hypervirulent K. pneumoniae (HvKp) |
| kp265 | + | + | + | + | + | + | Hypervirulent K. pneumoniae (HvKp) |
| kp351 | + | + | + | + | + | + | Hypervirulent K. pneumoniae (HvKp) |
| kp450 | + | + | + | + | + | + | Hypervirulent K. pneumoniae (HvKp) |
| kp506 | - | + | + | + | + | + | Hypervirulent K. pneumoniae (HvKp) |
| kp552 | + | + | + | + | + | + | Hypervirulent K. pneumoniae (HvKp) |
| kp680 | + | + | + | + | + | + | Hypervirulent K. pneumoniae (HvKp) |
| kp699 | + | + | + | + | + | + | Hypervirulent K. pneumoniae (HvKp) |
| kp707 | + | + | + | + | + | + | Hypervirulent K. pneumoniae (HvKp) |
| kp708 | + | + | + | + | + | + | Hypervirulent K. pneumoniae (HvKp) |
| kp2906 | - | + | + | + | + | + | Hypervirulent K. pneumoniae (HvKp) |
| kp192 | + | + | + | + | + | + | Hypervirulent K. pneumoniae (HvKp) |
| kp348 | + | + | + | + | + | + | Hypervirulent K. pneumoniae (HvKp) |
| kp614 | + | + | + | + | + | + | Hypervirulent K. pneumoniae (HvKp) |
| kp539 | + | + | + | + | + | + | Hypervirulent K. pneumoniae (HvKp) |
| kp65 | - | + | + | + | + | + | Hypervirulent K. pneumoniae (HvKp) |
| kp110 | + | + | + | + | + | + | Hypervirulent K. pneumoniae (HvKp) |
| kp137 | - | + | + | + | + | + | Hypervirulent K. pneumoniae (HvKp) |
| kp167 | + | + | + | + | + | + | Hypervirulent K. pneumoniae (HvKp) |
| kp184 | - | + | + | + | + | + | Hypervirulent K. pneumoniae (HvKp) |
| kp373 | + | + | + | + | + | + | Hypervirulent K. pneumoniae (HvKp) |
| kp398 | - | + | - | + | + | + | Hypervirulent K. pneumoniae (HvKp) |
| kp299 | + | + | + | + | + | + | Hypervirulent K. pneumoniae (HvKp) |
| kp105 | + | - | + | + | - | + | Hypervirulent K. pneumoniae (HvKp) |
| kp464 | - | - | - | - | - | - | Classical K. pneumoniae |
| kp82 | - | - | - | - | - | - | Classical K. pneumoniae |
| kp154 | - | - | - | - | - | - | Classical K. pneumoniae |
| kp466 | - | - | - | - | - | - | Classical K. pneumoniae |
| kp105 | + | + | - | + | - | + | Classical K. pneumoniae |
| kp109 | + | + | - | + | - | + | Classical K. pneumoniae |
| kp324 | - | - | - | - | - | - | Classical K. pneumoniae |
| kp517 | - | - | - | - | - | - | Classical K. pneumoniae |
| kp621 | - | - | - | - | + | - | Classical K. pneumoniae |
| kp610 | - | - | - | - | + | - | Classical K. pneumoniae |
| kp266 | - | + | - | - | - | - | Classical K. pneumoniae |
| kp94 | - | - | - | - | - | - | Classical K. pneumoniae |
| kp325 | - | - | - | - | - | - | Classical K. pneumoniae |
| kp331 | - | - | - | - | - | - | Classical K. pneumoniae |
| kp202 | - | - | - | - | - | - | Classical K. pneumoniae |
| kp27 | - | - | - | - | - | - | Classical K. pneumoniae |
| kp43 | - | - | - | - | - | - | Classical K. pneumoniae |
| kp166 | - | - | - | + | + | + | Classical K. pneumoniae |
| kp199 | - | - | - | - | - | - | Classical K. pneumoniae |
| kp328 | - | - | - | - | + | - | Classical K. pneumoniae |
| kp605 | - | - | - | - | + | - | Classical K. pneumoniae |

Isolates were aligned according to bacterial species and virulence.

^a^Bacterial species are discribed according to molecular identification. 'HvKp' represents that the isolate is hypervirulent with the definition employed in this study.

^b^If a virulence gene cluster was partially possessed, the part of the gene identified was described.
