# Supplementary material for: Clinical and Molecular Characteristics and Antibacterial Strategies of Klebsiella pneumoniae in Pyogenic Infection
Source: Microbiol Spectr. 2023 Jun 21;11(4):e00640-23. doi: 10.1128/spectrum.00640-23 (PMC10434161; doi:10.1128/spectrum.00640-23)
Supplement: Supplemental file 4 — Table S2. Download spectrum.00640-23-s0004.docx, DOCX file, 0.03 MB [file spectrum.00640-23-s0004.docx]

Table S2 Drug sensitivity test of 8 strains of K. pneumoniae.

| **Strain  No.** | **Bacterial  species** | **Capsular  genotype** | **string  test** | **Sequence  type** | **rmpA** | **CIP** | | **LEX** | | **PMB** | | **GEN** | | **CRO** | | **CTX** | | **MEM** | | **IPM** | |
| --- | --- | --- | --- | --- | --- | --- | --- | --- | --- | --- | --- | --- | --- | --- | --- | --- | --- | --- | --- | --- | --- |
|  |  |  |  |  |  | **MIC** | **MBC** | **MIC** | **MBC** | **MIC** | **MBC** | **MIC** | **MBC** | **MIC** | **MBC** | **MIC** | **MBC** | **MIC** | **MBC** | **MIC** | **MBC** |
| **kp27** | **cKp** | **cKp** | **<5mm** | **ST36** | **Neg** | **≤0.06** | **0.25** | **≤0.12** | **0.5** | **≤0.12** | **0.5** | **1** | **16** | **≤0.12** | **0.5** | **≤0.12** | **0.5** | **≤0.12** | **0.5** | **≤0.12** | **0.5** |
| **kp43** | **cKp** | **cKp** | **<5mm** | **ST3658** | **Neg** | **≤0.06** | **0.25** | **≤0.12** | **0.5** | **≤0.12** | **0.5** | **0.5** | **2** | **≤0.12** | **0.5** | **≤0.12** | **0.5** | **≤0.12** | **0.5** | **0.25** | **0.5** |
| **kp116** | **HvKp** | **K1** | **>5mm** | **ST23** | **Pos** | **≤0.06** | **0.25** | **≤0.12** | **2** | **≤0.12** | **8** | **0.5** | **8** | **≤0.12** | **4** | **≤0.12** | **4** | **≤0.12** | **4** | **0.5** | **1** |
| **kp114** | **HvKp** | **K1** | **>5mm** | **ST23** | **Pos** | **≤0.06** | **1** | **≤0.12** | **2** | **≤0.12** | **8** | **0.5** | **4** | **≤0.12** | **1** | **≤0.12** | **1** | **≤0.12** | **1** | **≤0.12** | **1** |
| **kp65** | **HvKp** | **K2** | **>5mm** | **ST65** | **Pos** | **0.12** | **2** | **≤0.12** | **2** | **≤0.12** | **1** | **1** | **16** | **≤0.12** | **4** | **≤0.12** | **1** | **≤0.12** | **2** | **≤0.12** | **1** |
| **kp110** | **HvKp** | **K2** | **>5mm** | **ST65** | **Pos** | **≤0.06** | **1** | **≤0.12** | **4** | **≤0.12** | **8** | **0.5** | **16** | **0.25** | **4** | **≤0.12** | **4** | **≤0.12** | **2** | **≤0.12** | **2** |
| **kp184** | **HvKp** | **K16** | **>5mm** | **ST660** | **Pos** | **0.5** | **8** | **0.5** | **8** | **1** | **8** | **2** | **16** | **0.25** | **8** | **0.25** | **4** | **≤0.12** | **2** | **≤0.12** | **4** |
| **kp373** | **HvKp** | **K16** | **>5mm** | **ST660** | **Pos** | **≤0.06** | **0.25** | **≤0.12** | **2** | **≤0.12** | **8** | **0.5** | **16** | **≤0.12** | **4** | **≤0.12** | **4** | **≤0.12** | **2** | **≤0.12** | **4** |
| **ATCC25922** | ***Escherichia coli*** | |  |  |  | **≤0.06** | **NT** | **≤0.12** | **NT** | **≤0.12** | **NT** | **0.5** | **NT** | **≤0.12** | **NT** | **≤0.12** | **NT** | **≤0.12** | **NT** | **≤0.12** | **NT** |

Six representative hvKp and two cKp strains were randomly selected from the clinical strains; CIP, Ciprofloxacin; LEX, Levofloxacin; PMB, polymyxin; GEN, gentamicin; CRO, ceftriaxone; CTX, cefotaxime; MEM, meropenem; IMP, imipenem; MIC, minimal inhibit concentration; MBC, minimum bactericidal concentration; cKp, Classic *Klebsiella pneumoniae*; HvKp, hypermucoviscous *Klebsiella pneumoniae*.
