# Supplementary material for: Clinical and Molecular Characteristics and Antibacterial Strategies of Klebsiella pneumoniae in Pyogenic Infection
Source: Microbiol Spectr. 2023 Jun 21;11(4):e00640-23. doi: 10.1128/spectrum.00640-23 (PMC10434161; doi:10.1128/spectrum.00640-23)
Supplement: Supplemental file 5 — Legends to Fig. S1 and S2. Download spectrum.00640-23-s0005.docx, DOCX file, 0.02 MB [file spectrum.00640-23-s0005.docx]

**Suppl supplementary material figure legends**

**FIG S1. Virulence gene profiles of 54 clinical Klebsiella pneumoniae isolates.**

**FIG S2. Drug resistance gene profiles of 54 clinical Klebsiella pneumoniae isolates.**
